# Neurophysiological mechanisms of optimized graphomotor performance in biscriptuals

**DOI:** 10.1162/IMAG.a.1265

**Published:** 2026-06-05

**Authors:** Yitong Zuo, Gaëlle Alhaddad, Víctor J. López-Madrona, Laure Spieser, Antonella Iannotta, Jean-Claude Gilhodes, Benjamin Morillon, Marieke Longcamp

**Affiliations:** Aix Marseille Université, CNRS, CRPN, Centre de Recherche en Psychologie et Neurosciences, Marseille, France; Aix Marseille Université, INSERM, INS, Institut de Neurosciences des Systèmes, Marseille, France; Laboratory of Functional Anatomy, Faculty of Human Motor Sciences, Université libre de Bruxelles (ULB), Brussels, Belgium; Laboratoire de Neuroanatomie et Neuroimagerie translationnelles, UNI – ULB Neuroscience Institute, Université libre de Bruxelles (ULB), Brussels, Belgium

**Keywords:** EEG, neural oscillations, predictive coding, biscriptuality, handwriting, graphomotor control

## Abstract

Many literate adults master two writing systems: a phenomenon termed biscriptuality. Biscriptuals do not just juggle two scripts—their graphomotor coordination also generally outperforms that of monoscriptuals, suggesting that some components of their motor system have been optimized. To uncover the neurocognitive foundations of this biscriptual advantage, we synchronized a loop-tracing task on a digitizing tablet with electroencephalographical (EEG) recordings. Theta (4–7 Hz) and beta (13–30 Hz) oscillatory dynamics were analyzed to test whether biscriptuals display better optimized planning and monitoring, and/or better sensorimotor control. At the behavioral level, biscriptuals displayed a robust advantage in tracing frequency and stability compared to monoscriptuals. At the neural level, biscriptuals showed lower frontal theta power than monoscriptuals, with frontal theta power positively correlated with behavioral variability. Optimization of predictive inference mediated by midfrontal theta oscillations thus stands out as a hallmark of the biscriptual advantage. Biscriptuals also displayed a lower degree of beta synchronization over parieto-occipital electrodes than monoscriptuals, with beta power being less correlated with kinematic dynamics. Similar effects in the beta band were observed for all participants when task difficulty decreased. These effects suggest higher reliance on sensory feedback when graphomotor execution is more demanding. Overall, these results call for a model of handwriting control where prefrontal and sensorimotor components contribute to implementing internal predictive models, whose accuracy and reliability depend on the type of expertise acquired when learning to write.

## 1 Introduction

Handwriting requires a sophisticated coordination of the fingers and the wrist, together with the dynamic application of force to hold the pen and progress in a specific direction. It is a movement of remarkable finesse and complexity. While Latin-based scripts dominate the scientific approaches of writing, there is, in fact, a multitude of graphic systems with a high degree of variation in their shape and linguistic dimensions. With globalization and migrational movements, an increasing amount of non-latin literate populations are learning and using two scripts in their daily lives. This phenomenon is termed biscriptuality (Usanova, 2019; Vaid, 2022). Biscriptuality offers a unique opportunity to understand the processes that allow the remarkable fluency of handwriting movements.

In previous studies (Alhaddad et al., 2023, 2024), we investigated whether Latin-Arabic biscriptuality acquired early in childhood has an influence on graphomotor performance. We designed a task where participants traced continuous loops in two writing conditions (left-to-right and right-to-left). This task efficiently probes the graphomotor dynamics of handwriting. According to the dynamic patterns theory, graphomotor dynamics result from the nonlinear coupling of two oscillatory movements in the orthogonal plane, which are executed by the wrist and the fingers respectively (Athènes et al., 2004; Danna et al., 2011; Hollerbach, 1981; Kelso et al., 1998; Sallagoïty et al., 2004). Per this approach, different graphomotor coordination patterns emerge from modulations of the amplitude, frequency, and phase of the oscillators, with the difference between two phases defining the relative phase (RP; Haken et al., 1985; Kay, 1988). Different relative phases lead to different degrees of eccentricity in the elliptical shapes formed by the coupling of the two oscillators (0° RP would correspond to a line and 90° RP to a circle; Athènes et al., 2004; Sallagoïty et al., 2004). RP and its standard deviation of RP (RPsd) have been related to the expertise of the graphomotor behavior. Indeed, expert adults who have largely automatized this behavior show a preference of 45° for the RP when asked to produce spontaneous ellipses, whereas children trace rounder patterns with an RP of roughly 90° (Athènes et al., 2004; Danna et al., 2012). Adults are more inclined to trace rounder patterns closer to those of children when more difficult conditions impose additional constraints (Danna et al., 2011, 2012). Additionally, in a series of loops, the variability of the relative phase, indexed by RPsd, reflects the similarity of their degrees of eccentricity and shapes, therefore the stability of the movement (Athènes et al., 2004). Finally, the frequency of the oscillators typically decreases with the difficulty of execution and increases with expertise (Danna et al., 2011, 2012). Collectively, these measures index graphomotor coordination performance. In our previous studies, biscriptuals displayed a higher spontaneous tracing frequency and a smaller RPsd than monoscriptuals, while both groups displayed a similar preference for the left-to-right condition (Alhaddad et al., 2023, 2024). We, thus, argued for an advantage of biscriptuals over monoscriptuals in graphomotor coordination. While this empirical observation supports the idea that the simultaneous and decade long training in two distinct scripts shapes the organization of manual actions, the mechanisms underpinning this advantage remain a fully open question.

The planning and control of handwriting can be viewed as relying on the implementation of internal predictive models built from the specification of expected outcomes of the action within the sensorimotor system (Danna et al., 2022; Grossberg & Paine, 2000; Wolpert & Ghahramani, 2000). The so-called inverse models use the desired and actual position of the pen as inputs to estimate the necessary motor command then inverted through Bayesian inference to optimize actions (Grossberg & Paine, 2000; McNamee & Wolpert, 2019; Mussa-Ivaldi & Bizzi, 2000). On the basis of a copy of this motor command (the efference copy), the forward model predicts the sensory consequences of the pen movement in order to compare them with the actual sensory feedback, or to attenuate the reliance on sensory feedback depending on the confidence associated with the prediction (Miall & Wolpert, 1996; Wolpert & Ghahramani, 2000). The neural underpinnings known for handwriting are compatible with this account of graphomotor control. For instance, the cerebellum and the posterior parietal lobes, whose fundamental contribution to several aspects of internal sensorimotor models has been emphasized (Kawato, 1999; McNamee & Wolpert, 2019), are two major nodes of the network underlying writing (Palmis et al., 2017; Planton et al., 2013). Other regions of the sensorimotor system such as the primary motor cortex and dorsal premotor cortex have also been reliably evidenced in studies of handwriting production (Planton et al., 2013). Accurate implementation of internal models is thought to rely upon efficient probabilistic inference integrating incoming sensorimotor information with prior contextual knowledge (Körding & Wolpert, 2006). The computation of the intended action outcome and the updating of the priors in the case of prediction error is thought to rely largely upon the prefrontal cortex, considered as a key element for this probabilistic inference. Recently, some researchers have emphasized the need for a common framework for interpreting the variety of functions assigned to the frontal lobes, usually grouped under the label “cognitive or executive control” (Alexander & Brown, 2018; Alexander et al., 2018; Soltani & Koechlin, 2022). They argued that the internal predictive models framework may be extended into the prefrontal cortex, where predictive models of stimulus and action outcomes are combined to contextual models of states of the environment to provide task sets that drive behavior (Soltani & Koechlin, 2022), a process that is critical to adequately plan and monitor graphic behaviors. In addition, handwriting being a continuous action, the prediction needs to be constantly updated during actual tracing, pointing to an important reliance on prefrontal mechanisms allowing working memory maintenance (Grossberg & Paine, 2000). The prefrontal control system is strongly recruited during learning how to write and remains involved in experts (Alhaddad et al., 2025; Palmis et al., 2021), and sulcal patterns in the anterior cingulate cortex (ACC) predict graphomotor performance in both learners and experts (Cachia et al., 2025). This highlights the importance of the functional mechanisms and the anatomical features of the prefrontal cortex in graphomotor control.

In this framework, the biscriptual advantage may indicate that the predictive inference processes and internal models implemented to plan and execute handwriting are more accurate in biscriptuals than in monoscriptuals. Both the prefrontal control system and the sensorimotor system are, thus, likely to play a role in the emergence of the biscriptual advantage. The study of biscriptuality, thus, provides a unique opportunity to advance our knowledge on the neural organization of handwriting and its different types of expertise. Here, we aimed at uncovering the mechanisms underlying the biscriptual advantage by considering an internal predictive model account of graphomotor behavior, implemented in the brain as oscillatory neural activity in different frequency bands. This approach is further justified by the nature of graphomotor coordination, an intrinsically rhythmic behavior (Hollerbach, 1981; Nutt, 1917). Among the oscillatory dynamics supporting predictive processes across cortical regions, theta and beta activities appear particularly relevant. Theta oscillations are highly prevalent in the frontal lobe and assumed to be the temporal templates that organize midfrontal predictive processes involved in action planning (Cavanagh & Frank, 2014; Keitel & Gross, 2016). In motor experts, theta oscillations contribute to the integration of acquired prior knowledge to compute forward models, thereby mediating predictive accuracy (Wilken et al., 2025a, 2025b). Beta activities, on the other hand, have been associated with the weighting between sensory afference and internal forward predictions (C. E. Palmer et al., 2019). They integrate preceding prediction errors, and current sensory uncertainty about the state of the body and the environment, in order to update or maintain the ongoing motor command (Jahani et al., 2020; C. Palmer et al., 2016; Tan et al., 2016). We, thus, coupled a previously developed loop-tracing task (Alhaddad et al., 2023, 2024) with electroencephalographic (EEG) recordings. We analyzed the dynamics of oscillatory activity and their relationship with behavioral outcomes of the task. As in the original Alhaddad et al. (2023)’s study, we also manipulated task difficulty, operationalized as the tracing condition (left-to-right: easy vs right-to left: difficult), in order to distinguish potentially specific effects of expertise from more general effects of task difficulty.

We indexed the activity of the prefrontal control system with midfrontal theta dynamics. Theta synchronization typically increases with greater task demands and is associated with behavioral variability (Watanabe et al., 2021; Cooper et al., 2017). Theta has been shown to be phased-locked to the onset of the first stroke during handwriting, this phenomenon being influenced by the level of mastery of the task (Pei et al., 2021). Cortico-cortical coherence of oscillations in the theta range encompasses distinct functional elements that support different variants of handwriting and is modulated by task modalities (Saarinen et al., 2020). In parallel, we indexed the efficiency of the sensorimotor system for the implementation of internal models with beta oscillation dynamics. A sustained beta desynchronization over the central region indexes smooth control of the tracing movements (Hervault et al., 2021; Lebar et al., 2017), and the amplitude of beta desynchronization is related to task difficulty during motor tasks (Chung et al., 2017; Watanabe et al., 2021). Consistent with its assumed role in predictive motor control, the level of beta oscillations during a continuous motor task is known to be associated with the motor output (Lv et al., 2010; Yuan et al., 2010), and with sensory processes. For instance, they are functionally relevant to discrete perception of somatosensory input (Baumgarten et al., 2015), and support the visual information processing in the magnocellular-dorsal pathway (Di Dona & Ronconi, 2023).

Therefore, theta oscillation dynamics and their correlation with behavioral variability should differentiate mono- from biscriptuals if, indeed, the biscriptual advantage is driven by functional differences in the predictive inferences computed in the prefrontal cortex. Instead, beta oscillation dynamics and their correlation to pen-position dynamics should differentiate mono- from biscriptuals if indeed the biscriptual advantage is driven by functional differences in the implementation of internal models in the sensorimotor system. Finally, if these effects are specifically driven by writing expertise, they should differ from effects of task difficulty (writing condition).

## 2 Methods

### 2.1 Participants

Thirty-one right-handed biscriptual and 31 age- and gender-matched monoscriptual participants were recruited for the experiment.

All subjects met the following criteria: a) be aged between 18- and 30-years old, b) be right-handed, c) have a higher level of education (having obtained a high-school diploma and attended university-level courses), and d) for biscriptual participants, speak fluently and write with equal proficiency in French and Arabic, all biscriptual participants had to be native Arabic speakers and writers. The demographic information of the subjects is shown in Table 1.

**Table 1.** Sample characteristics, writing habits, academic and linguistic backgrounds of the two groups of participants.

	Monoscriptuals (n = 31)	Biscriptuals (n = 31)
Age	23 (range: 19 - 29)	23 (range: 18 - 33)
Gender		
Male	40.62%	41.93%
Female	59.37%	58.06%
Level of education		
BSc	34.37%	32.25%
MSc	43.75%	51.61%
PhD	21.87%	16.12%
Writing habits		
Number of pages written in Latin script per week		
<5	56.26%	42.28%
≥5	43.74%	57.72%
Number of pages written in Arabic script per week		
<5	N/A	83.85%
≥5	N/A	16.15%
Typing habits		
Number of pages typed in Latin script per week		
<5	40.64%	22.59%
≥5	59.36%	77.41%
Number of pages typed in Arabic script per week		
<5	N/A	100%
≥5	N/A	
Alphabet used when typing in Arabic		
Arabic only	N/A	9.67%
Latin only	N/A	48.38%
Latin and Arabic	N/A	41.93%
Spoken languages		
L2 English	100%	0%
French	N/A	100%
L2 AOA		
<4	0%	80.50%
4–9	37.53%	19.50%
≥10	62.47%	0%
Number of years in France		
<5	N/A	83.88%
≥5	N/A	16.12%
Subjective assessment of financial situation		
Financially insecure	13.53%	9.93%
Somewhat financially secure	52.48%	49.60%
Financially secure	33.99%	40.47%

L2 = Second Language; AOA = Age of Acquisition.

Participants reported no relevant medical history, had normal or corrected-to-normal vision, and signed a written informed consent before the experiment. The experimental study was approved by the local ethics committee.

As a way of controlling for factors that could affect graphomotor control, we administered the LEAP-Q questionnaire (Marian et al., 2007) that identified the spoken languages and written alphabets of the participants through questions that assessed the duration, amount, and frequency of exposure to French and Arabic as well as their manual handedness. We also administered a questionnaire about their socio-economic status, knowing that these aspects play an important role on handwriting proficiency (Albaret et al., 2013). Another questionnaire on writing habits was also administered. Each participant received 30€ as financial compensation. Six participants were excluded from the final dataset, with three containing more than 25% of noisy electrodes, one for whom sufficient interpolation could not be performed after channel rejection, one failing to follow the instructions, and one with corrupted behavioral data.

### 2.2 Kinematic data acquisition

The handwriting movements were recorded with a 16” Wacom Cintiq® Screen tablet connected to a computer via a USB cable and its specific ballpoint pen. This tablet has a sampling frequency of 180 Hz. The kinematic data were recorded and synchronized with the EEG recording through a program running in PsychoPy (Peirce et al., 2019; version: PsychoPy Builder v2022.2.5). This allows the setup to send triggers both when the stimuli appear on the screen and when the participants’ pen first comes in contact with the screen to start tracing.

### 2.3 EEG data acquisition

EEG data were recorded using 64 Ag/AgCl surface electrodes positioned according to the 10/20 International System and connected to a BioSemi ActiveTwo AD-Box amplifier model. The signals were digitized at a sampling rate of 512 Hz (Actiview acquisition program). Three Ag/AgCl electrodes placed near the canthus of each eye and under the left eye orbital allowed us to record blinks and horizontal eye movements. Two external electrodes were placed bilaterally on the mastoids.

### 2.4 Task and procedure

Participants were seated on a chair in a dimly lit Faraday cage and were instructed to trace loops on the digitizing tablet with their right (dominant) hand. Prior to the experimental task, participants were instructed to adjust the positions of the tablet and the chair to their personal comfort, in order to minimize head and body movements that could disrupt the EEG recordings.

**Fig. 1.**
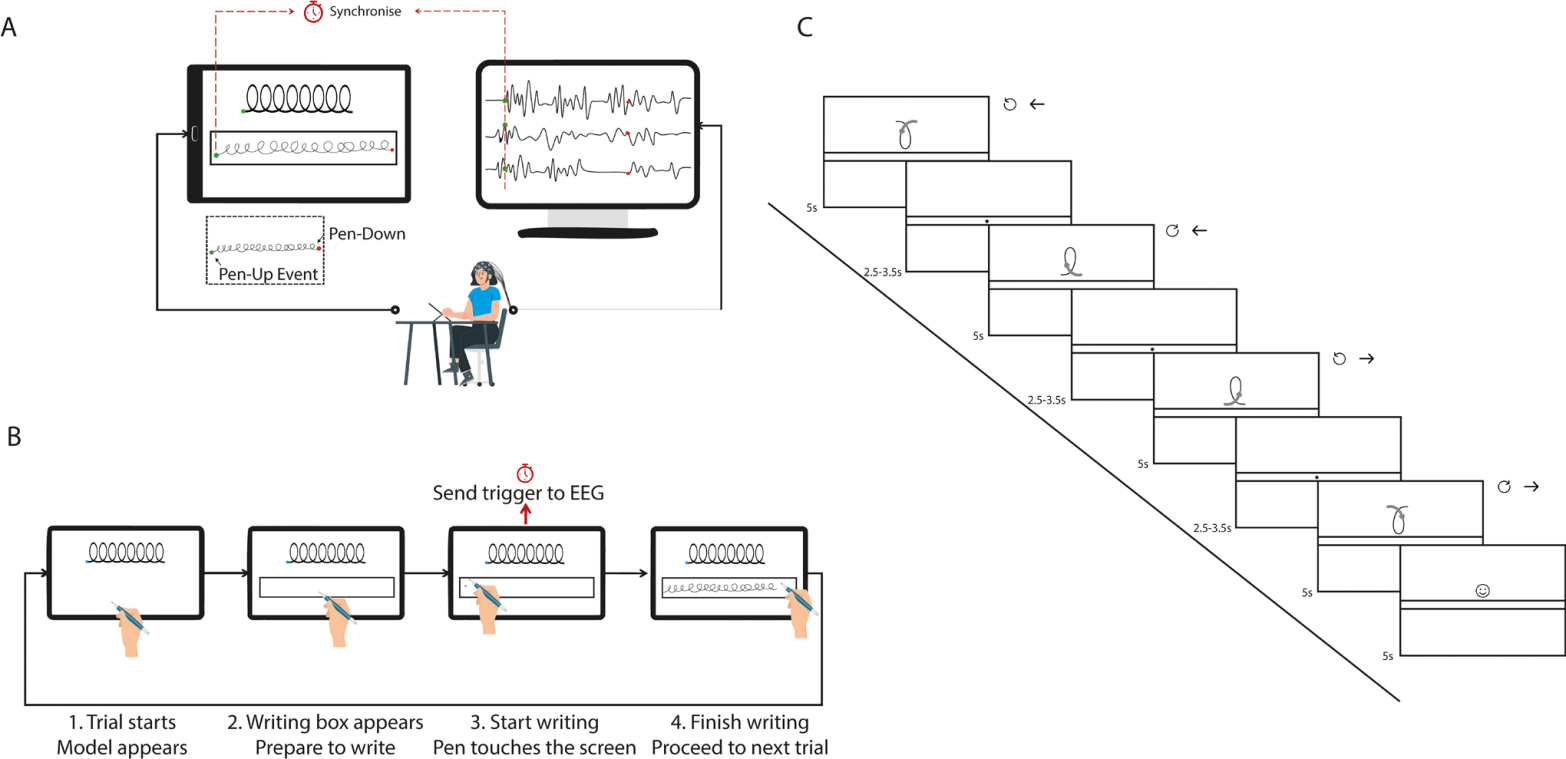
Experimental design. (A) Experimental setup: loop tracing task on a digitizing tablet synchronized with the EEG recording system (adapted from Pei et al., 2021). (B) Example of a structure of one trial (left-to-right condition). (C) Example of the four possible stimuli (left to- right downward and upward, and right-to- left upward and downward) and a blinking period.

The framework for this study was built upon the work of Alhaddad et al. (2023). We adjusted the loop-tracing task to comply with the constraints of EEG recordings. To minimize eye movements, we positioned the stimuli in the central vision field of participants on the screen and provided clear tracing guidelines. The experiment consisted of four blocks, each containing 52 trials and five blinking periods. Each trial began with the presentation of two lines, between which participants were required to trace (Fig. 1B). The instructions mentioned that the lines were more a guideline than a constraint, so that the tracing could extend beyond the lines. A fixation cross then appeared in the middle, between the two lines. Participants were instructed to keep their pen tip near the fixation cross without touching the tablet, triggering the appearance of stimuli (loop condition) above the upper line of the tracing area. Once the stimuli appeared, participants started tracing continuous loops based on the stimulus. Two trials belonged to either of two conditions: left-to-right or right-to-left (Fig. 1C). In each of the 2 conditions, loops could be traced upwards or downwards. The tracing duration for each trial was set to 5 seconds, followed by a random inter-trial interval of 2.5, 3, or 3.5 seconds before the next trial commenced (Fig. 1C).

Given the task’s lengthy nature, we incorporated blinking periods represented by a smiley face emoticon. During these periods, participants could blink and make minor movements in facial, neck, and upper body muscles without significant movements. Ample time was allocated for participants to relax between blocks. Trial orders within blocks, the length of intervals between trials, and the sequence of blocks among participants were randomized using the Mix and Match program (van Casteren & Davis, 2006). The criterion for the randomization was that no same condition nor same inter-trial interval should be repeated more than twice in a row, in order to avoid anticipation.

## 3 Data Pre-Processing

### 3.1 Kinematic data

Loop series that were traced in the wrong condition, that were not continuous (presence of pen lifts) or that were too angular (thus affecting behavioral values) were removed. In total, 316 trials were removed from a total of 13104 loops series that were traced by participants (2.41%). The manual inspection and the following extraction of parameters were done with a software based on Java developed in the laboratory.

### 3.2 EEG data

We first matched the trials registered in the EEG data and the kinematic behavioral data through custom Matlab scripts. Reaction time for each trial was calculated as the delay between the trigger representing the moment where the stimulus appears and the trigger representing the moment where the pen first touches the screen. We then removed the epochs whose reaction times exceeded 2000 ms or were below 100 ms. These procedures resulted in a total exclusion of 2.56% of all trials. EEG pre-processing was then performed using the Brainstorm toolbox (Tadel et al., 2011) and custom MATLAB scripts. After adding the channel positions (BioSemi 64 10-10 system), initial Power Spectrum Density (PSD) was calculated over the entire recordings for each participant (Welch’s method, with the window length of 4000 ms and the window overlap ratio of 50%), as means to reject noisy electrodes by visually inspecting them. This resulted in an average exclusion of 2.80 (SD = 2.36) electrodes for each participant. EEG data were then re-referenced to average and high-pass filtered at 0.1 Hz. Independent component analysis (ICA) was performed over the entire recordings with the Picard function to remove artifacts caused by ocular movements. The removal of ICA components implicates only blinks and horizontal ocular saccades (Chaumon et al., 2015). On average 1.85 (SD = 0.36) independent components were removed for each participant. We then interpolated electrodes that were excluded during the initial PSD analysis with the maximum distance of 5 cm between neighbours. Finally, we cut the recordings into epochs from -3000 to +7000 ms around stimulus onset. Since the trials last 5000 ms after the stimulus onset, the epoch window of -3000 to +7000 ms covers the entire period of stimulus processing, motor planning as well as motor execution. The supplementary 1000 and 2000 ms included before the start of the window used to calculate PSDbaseline (a window of 2000 ms before the stimulus onset, see Power Spectrum Density Analysis) and after the end of the tracing assure that the edge effects in the subsequent time-frequency decomposition are not calculated on the target data to be analysed and can be discarded. Since our frequency bands of interest has a lower threshold of 4 Hz, these extra segments can largely cover at least 4 cycles of the slowest oscillation of interest, guaranteeing a non-contamination of these effects (Cohen, 2014).

## 4 Data Processing

### 4.1 Kinematic data

We computed the same variables as in Alhaddad et al. (2023) using partly different methods to accommodate the large data set.

The tracing signal was recorded at 180 Hz. The power spectral density of the filtered signal in the vertical dimension was estimated using a Fast Fourier Transform (FFT). The first frequency peak identified in the spectrum, corresponding to the dominant frequency component, was extracted and used as the loops’ frequency measure, expressed in Hz (Issartel et al., 2015).

**Fig. 2.**
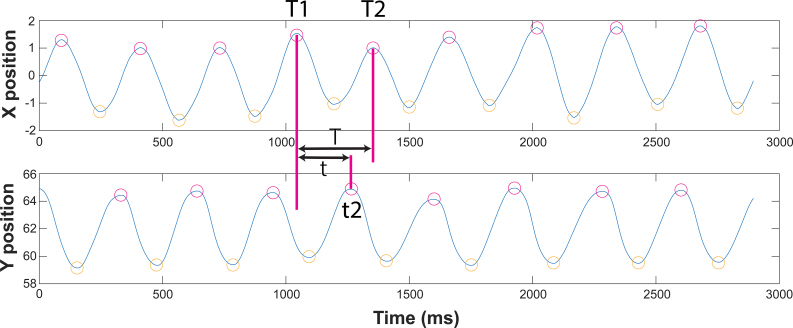
Schematic presentation of the calculation of the relative phase. The oscillatory profiles of the pen-tip position in the X and Y dimension are shown on top and bottom and serve as reference and target profiles respectively. Purple circles depict the maxima and the orange circles correspond to the minima. Here, we use the maxima as example for the calculation. T represents a full cycle from the reference profile, and t represents the time difference between the subsequent peak in the target profile and the first peak in the reference profile. The relative phase corresponds therefore to the percentage of t over T.

RP and RPsd were calculated using the point estimation method (Zanone & Kelso, 1997). This method is based on the detection of precise points, such as the maxima (peaks) or minima (reversals in direction) of segment motion to estimate the timing relationship between two oscillating segments (Galgon & Shewokis, 2016). From the moments of maxima (peaks) and minima (reversals in direction), discrete relative phase is calculated by the following formula (Fig. 2):

$$\theta =\left({\text{t}}_{2}\;-{\text{T}}_{1}\right)/\left({\text{T}}_{2}\;-{\text{T}}_{1}\right)\times 360=\left(\text{t}/\text{T}\right)\times 360$$

Where T1 represents a turnaround point (minimum or maximum) in the oscillatory profile serving as reference, and t2 corresponds to a turnaround point in the oscillatory profile serving as target, and T2 points to the next turnaround point in the reference profile. This discrete method was opted since it measures the RP between any type of periodic oscillators, regardless of the shape and the characteristics of their cycles, and it provides a high temporal precision (Zanone & Kelso, 1997).

We also centered phase values to ensure directional consistency in cyclic phase data (Venkatasubramanian et al., 2016), values of relative phase greater than 180° were transformed using the formula 360—phase relative value. This adjustment reflects the phase angle while preserving its directional tendencies, allowing all values to be expressed within a 0–180° range for clearer interpretation (Athènes et al., 2004).

The resulting behavioral data were analyzed using Linear Mixed Models. This allowed us to compare the graphomotor performance data between two groups (monoscriptuals and biscriptuals) in both writing conditions while controlling for random effect of participants using the following mixed effects formula: lmer(dependent variable ~ group*condition + age + gender + block_order + (1|subject), dataset). Model selection was performed using Akaike’s Information Criterion (AIC) (Chakrabarti & Ghosh, 2011). For all three dependent measures: frequency, RPsd, and mean RP, the model with the best fit was the linear mixed-effects model of the form: lmer(dependent variable ~ group × condition + (1 | subject), dataset). These models are reported in the Results section.

### 4.2 Power spectrum density analysis

EEG epochs were split according to the 2 tracing conditions (left-to-right and right-to-left), in line with the behavioral analysis. We computed the Power Spectrum Density (PSD) based on all trials of each tracing condition per participant (Welch’s method, with the window length of 4000 ms and the window overlap ratio of 50%). With the stimulus onset occurring at time 0, the PSDs were calculated from -2000 to 0 ms (PSDbaseline) and from 0 to 5000 ms (PSDtask). The length of PSDbaseline is smaller than the smallest inter-trial interval (2500 ms), ensuring that the baseline does not intrude into the previous epoch. We then computed the ratio of PSDtask over PSDbaseline, to normalize the task-related power spectral variations while minimizing the 1/f effect (Gyurkovics et al., 2022). We, therefore, obtained the ratio of PSDtask over PSDbaseline for all trials belonging to the same tracing condition for each participant, each channel and each frequency band (in 0.5 Hz steps) from 0 to 40 Hz.

Statistics were conducted via cluster-based non-parametric permutation test over all electrodes to test for the main effects of the group (monoscriptuals versus biscriptuals), condition (left-to-right versus right-to-left) and the interaction between group and condition, for each frequency band of interest, as implemented in the Fieldtrip function *ft_timelockstatistics* (Maris & Oostenveld, 2007). We used the temporal dimension in this function to represent the frequential dimension of our PSD ratio data. More specifically, this test compared averaged PSD ratio between groups, conditions and the difference between conditions for each group, for each electrode and each frequency band using a one-sided t-test (independent samples T-statistics for testing the main effect of group and the interaction of group and condition, and dependent samples T-statistics for testing the main effect of condition), resulting in one t-value for each channel and each frequency band. Frequency ranges were set to 4–7 Hz for theta oscillations and 13–30 Hz for beta oscillations per the commonly adopted canonical ranges, to capture the core frequential phenomenon associated with prefrontal control and sensorimotor mechanisms and to minimize overlaps with neighboring frequency bands (Buzsáki, 2006; Capilla et al., 2022; Cohen, 2014). It is to be noted that we have chosen a frequency window slightly larger (±1 Hz) than the a priori frequency ranges of interest (therefore 3–8 Hz for theta oscillations and 12–31 Hz for beta oscillations) to avoid boundary effect during cluster calculation. Restricting the computation to the original range might artificially truncate clusters that extend slightly beyond the predefined limits, thereby introducing bias in cluster size. Therefore, clusters were computed within the enlarged windows, while statistical interpretation was restricted to the frequency ranges of interest. We then searched for clusters within which at least two neighboring electrodes whose t-values meet the significant threshold (*p* < .05), and summed all the t-values within the significant cluster. Five thousand random partitions of the data were permuted for the cluster-level statistics to obtain a distribution, with which the probability of the significance of the cluster could be estimated (Cohen, 2014). Here we report all clusters that have a significance probability that meets the threshold (*p* < .05). We opted for this test because it allows correction for multiple comparisons, while permitting us to perform a whole-brain analysis including all electrodes, which is especially useful since beta oscillation dynamics are the vehicle of different cognitive functions in different regions. We visualized the significant clusters in topographical maps with the Fieldtrip function *ft_topoplotTFR*, demonstrating the channels and significant differences between groups or conditions.

### 4.3 Brain-behavior correlations

Since behavioral variability has been shown to be related to the level of theta power in previous studies (Cooper et al., 2017; Watanabe et al., 2021), we first tested he relationship between the theta power and the standard deviation of the relative phase of the loops (RPsd). Average PSD in the theta range (4-7 Hz) and RPsd were extracted for each trial and correlation analysis was calculated for all trials per subject. Trials containing only one loop were excluded from this set of analysis, since one loop can only yield one RP therefore no variability per se (5 trials in a total of 12774 trials, less than 0.04%). The correlation values were then compared to test values against zero to test the significance of the correlation, and the significant cluster of correlation values was visualized through a topographical map.

Second, we assessed the relationship between power in the beta range (13-30 Hz) and the position of the pen tip in the vertical dimension (y position) through a preliminary computation of the time-frequency decomposition (TF) resampled to 200 Hz for each trial. Behavioral data were also resampled to 200 Hz to match the TF. Beta activities might not be associated with discrete events such as the stimulus apparition or pen-down movement, but rather an ongoing dynamic state that co-varies with the continuously changing external variables, such as the visuospatial perception of the pen tip (Pei et al., 2021). Here we focus on y because it reflects the most accurately the movement frequency. The TFs and y positions were concatenated for each participant across all trials and conditions (208 trials per subject, the total number of all the trials) to test for the main effect of group, and for each tracing condition (104 trials per condition per subject, half of the total number of all trials) to test for the main effect of condition and the interaction of group and condition. This resulted in three sets of concatenated data for each subject. We then normalized all the concatenated data by subtracting with the mean (for TF this was done for each electrode and each frequency). Then, we cross-correlated TF and y position for each set of concatenated data, for each electrode and each frequency band, at different lags (±200 ms). To determine the significance of the correlation between TF and y position, we performed 1000 permutations. Within each permutation, the trial order of y position was randomized before concatenation and normalization, then cross-correlated with TF for each channel and each frequency. A cumulative distribution function based on the mean and standard deviation issued from the results of all the permutations was reconstructed for each lag, and the observed correlation values were compared against this distribution using a two-tailed threshold (*p* > .975 or *p* < .025) to determine their probability significance (see Supplementary Fig. S1 for a schematic presentation of the procedures to decide the significance of correlation on the single-subject level). Correlation values were then squared. Finally, we chose the maximal correlation values across all lags for each electrode, each frequency and each participant if they existed there. Statistical analysis utilized the same cluster-based non-parametric test to test the main effects of group, condition and the interaction of group and condition, and were conducted on data that had been pre-thresholded at the single subject level. Only the channel-frequency points that reached significance in the subject-level permutation test were retained; non-significant points were set to NaN. The comparison was done within the range containing one frequency step further from the two extremities of the beta range (13–30 Hz), in the same vein of the rationale for choosing a larger range for the statistical analysis for the PSD analysis, and only for the electrodes belonging to the significant clusters issued from the PSD results for the beta range. One monoscriptual participant was excluded for this specific analysis because their correlation value in the aforementioned frequency and channel range deviated from the group average by more than 3 standard deviations. Topographical representations of averaged correlation coefficients per group per condition, as well as of significant clusters were computed for visualization similarly as in the PSD analysis. In the resulting significant clusters, all the lags where the maximum correlation occurred were taken together for each group. For each subject, we performed a non-parametric Wilcoxon signed-rank test against zero with the approximate method for *p*-value computation, to assess whether the direction of the lag significantly deviated from zero at the single-subject level. This yielded a *p*-value and a z-value representing the significance of the condition of the lag distribution. We then used these z-values to describe the distribution of the lags across subjects. First, we assessed each group with the same non-parametric Wilcoxon signed-rank test, then we compared between groups using the Mann-Whitney U test. These tests do not assume normality of the distribution of the lags and allowed us to test whether each group showed a systematic lag direction and whether the lag direction differed significantly between groups.

## 5 Results

### 5.1 Biscriptuals are faster and more stable than monoscriptuals in loop tracing

Biscriptuals produced loops at a higher frequency than monoscriptuals (ß = -0.69, t = -4.01, *p* < .000; mean frequency: biscriptuals = 2.99 Hz, monoscriptuals = 2.32 Hz). Frequency was higher for the left-to-right than for the right-to-left condition (ß = 0.27, t = 25.98, *p* < .000; left-to-right = 2.80 Hz, right-to-left = 2.50 Hz). There was a main effect of the orders of blocks on frequency (ß = 0.04, t = 13.83, *p* < .000; block 1 = 2.57 Hz, block 2 = 2.65 Hz, block 3 = 2.66 Hz, block 4 = 2.71 Hz). Furthermore, there was a group by writing condition interaction (ß = 0.04, t = 3.23, *p* < .000; biscriptuals left-to-right = 3.13 Hz, biscriptuals right-to-left = 2.86 Hz, monoscriptuals left-to-right = 2.48 Hz, monoscriptuals right-to-left = 2.16 Hz). The interaction specifically revealed that the effect of condition was stronger in monoscriptuals (ß = -0.32, *p* < .000) than in biscriptuals (ß = -0.27, *p* < .000).

While the loops were produced with similar mean relative phase (RP) between monoscriptuals and biscriptuals (ß = 0.47, t = 0.17, *p* = .86), we observed a higher RP for right-to-left than left-to right condition (ß = -2.14, t = -6.56, *p* < .000; left-to-right = 83.34°, right-to-left = 86.38°). Both groups produced loops with an RP closer to 90° in the right-to-left conditions, while both groups traced loops with an RP closer to 45° in the left-to-right conditions. This indicates that right-to-left conditions imposed more difficulties for all participants, rendering their graphomotor pattern rounder and closer to a pattern preferred by individuals who have less expertise (Alhaddad et al., 2023; Danna et al., 2011). Results also indicated a group by writing condition interaction (ß = -1.62, t = -3.57, *p* < .000; biscriptuals left-to-right = 83.87, biscriptuals right-to-left = 86.06, monoscriptuals left-to-right = 82.85, monoscriptuals right-to-left = 86.67). This interaction indicated stronger effects of the more right-to-left conditions in monoscriptuals (ß = 3.76, *p* < .000) than in biscriptuals (ß = 2.14, *p* < .000). The effects of group and block order did not reach significance.

Finally, analysis of the standard deviation (SD) of the RP (RPsd) showed that biscriptuals produced more stable loops than monoscriptuals (ß = 3.41, t = 3.62, *p* < .000; biscriptuals = 15.95°, monoscriptuals = 18.30°). The RP was more variable in the right-to-left than in the left-to-right condition (ß = -0.52, t = -2.12, *p* = .003; left-to-right = 16.37°, right-to-left = 17.96°). There was a main effect of the orders of blocks on the loop stability (ß = -0.42, t = -5.44, *p* < .000; block 1 = 17.75°, block 2 = 17.39°, block 3 = 17.12°, block 4 = 16.44°). Finally, a group by writing condition interaction was significant (ß = -2.09, t = -6.07, *p* < .000; biscriptuals left-to-right = 15.70°, biscriptuals right-to-left = 16.20°, monoscriptuals left-to-right = 16.99°, monoscriptuals right-to-left = 19.62°). This indicated that the differences between the left-to-right and the right-to-left conditions were more pronounced in monoscriptuals than in biscriptuals. Contrast analysis indicated differences between the left-to-right and the right-to-left conditions in monoscriptuals (ß = 2.62, *p* < .000), as well as differences in the right-to-left condition between monoscriptuals and biscriptuals (ß = -3.41, *p* < .000).

**Fig. 3.**
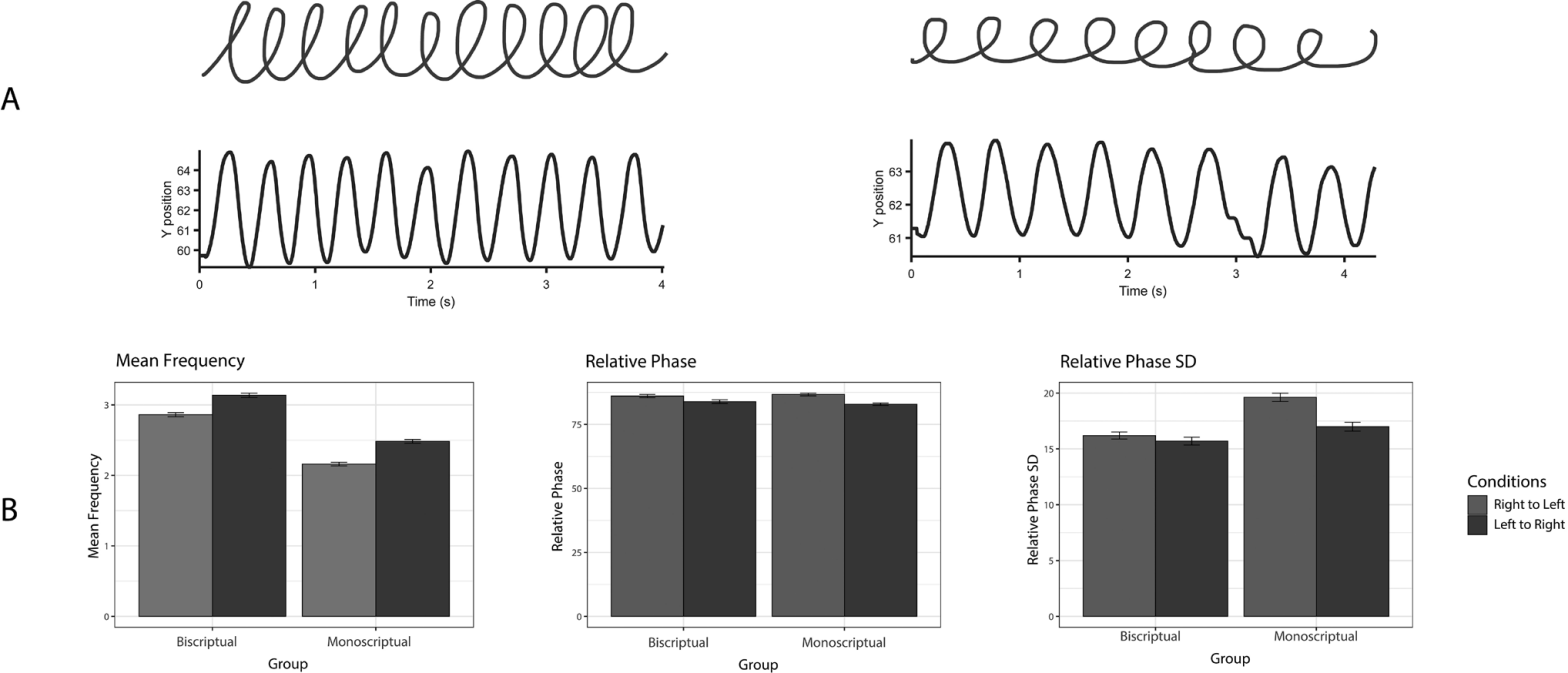
(A) Examples of loops and the corresponding y position of the pen tip of two trials from a biscriptual participant **(left)** and a monoscriptual participant **(right)**. The biscriptual participant traced with a higher frequency and a lower RPsd. (B) Effects of writing direction on tracing frequency, Relative Phase (RP) and standard deviation (SD) of the RP (RPsd) of loops in monoscriptual and biscriptual participants.

Overall, this shows that biscriptuals produce faster and more stable movements than monoscriptuals, with reduced sensitivity to writing condition effects (i.e., task difficulty). All participants produced less variable loops that contain graphomotor pattern more similar to that of expert writers, in a faster manner. The summary of the behavioral results is shown in Table 2 and Figure 3.

**Table 2.** Effects of writing condition and group on frequency, mean and standard deviation of the relative phase.

Frequency				
Fixed effects	ß	SE	t	*p*
(Intercept)	2.74	0.12	22.10	.000***
Group	-0.69	0.17	-4.01	.000***
Writing condition	0.27	0.01	25.98	.000***
Group x Writing condition	0.04	0.01	3.23	.001**
Mean RP				
	ß	SE	t	*P*
(Intercept)	86.57	2.01	42.88	.000***
Group	0.47	2.78	0.17	.864
Writing condition	-2.14	0.32	-6.56	.000***
Group x Writing condition	-1.62	0.45	-3.57	.000***
RPsd				
	ß	SE	t	*P*
(Intercept)	17.28	0.70	24.59	.000***
Group	3.41	0.94	3.62	.000***
Writing condition	-0.52	0.24	-2.12	.033*
Group x Writing condition	-2.09	0.34	-6.07	.000***

*p < .05, **p < .01, ***p < .001.

### 5.2 Stronger theta power in monoscriptuals reflects greater behavioral variability during loop tracing

**Fig. 4.**
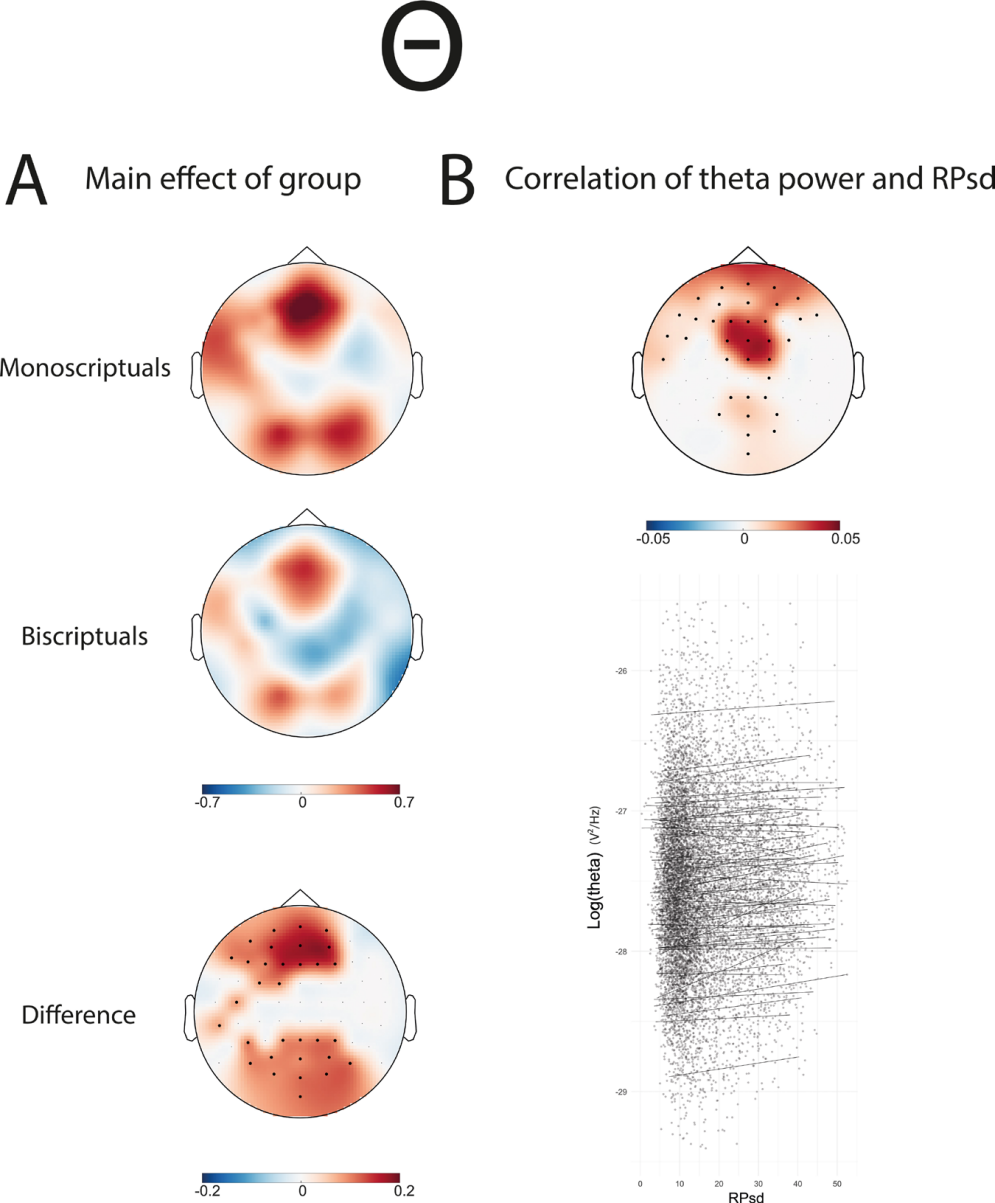
Theta power (4-7 Hz) during loops tracing. (A) Main effect of group on theta power. From top to bottom: Theta power averaged across monoscriptuals; Theta power averaged across biscriptuals; the significant cluster issued from the group comparison of theta power, with black dots denoting electrodes belonging to the cluster. (B) **Top:** topographical representation of correlation values between neural theta power and kinematic RPsd, estimated across groups, conditions and trials. Black dots indicate electrodes displaying significant correlation coefficients. **Bottom:** Visualisation of the data for the FCz electrode. Scatter plots of the RPsd and log transformed theta power (V²/Hz) for all trials, RPsd is on the x-axis and log-transformed theta amplitude of the FCz electrode is on the y-axis. A small constant (ε = 10^−20^) was added before the log transformation to avoid undefined values at zero. Each point represents a trial after outlier removal (trials whose RPsd or theta amplitude values deviated more than ±3 SDs from the group mean within each group were excluded).

Loop tracing was associated with an increase in theta-band power in both groups compared to pre-movement baseline, predominantly over frontal and occipital electrodes (Fig. 4A). A significant positive cluster emerged (*p* < .023) when monoscriptuals were compared with biscriptuals (i.e., group comparison), indicating a stronger theta increase in monoscriptuals. This cluster covered a wide range of frontal, parietal and occipital electrodes, with steeper effects in the frontal electrodes. There was no significant difference between conditions nor interaction effect of group and condition for theta power.

Brain-behavior correlation analysis focused on RPsd and theta-band power of each trial since RPsd represents the variability of the behavior, which has been previously linked to frontoparietal theta oscillations (Cooper et al., 2017). Theta power was positively correlated with the behavioral variability of loop tracing (RPsd). Because RPsd captures intra-individual variability in motor stability, data from all groups and conditions were pooled for this analysis. A significant cluster (*p* < .001) emerged when correlation coefficients were compared to zero, centred over midfrontal electrodes. For visualisation purposes, the data of the FCz electrode are displayed (Fig. 4B, bottom). Overall, this shows that frontal theta power is positively correlated with motor variability during loop tracing.

### 5.3 Stronger occipital beta synchronization and beta–movement coupling in monoscriptuals

**Fig. 5.**
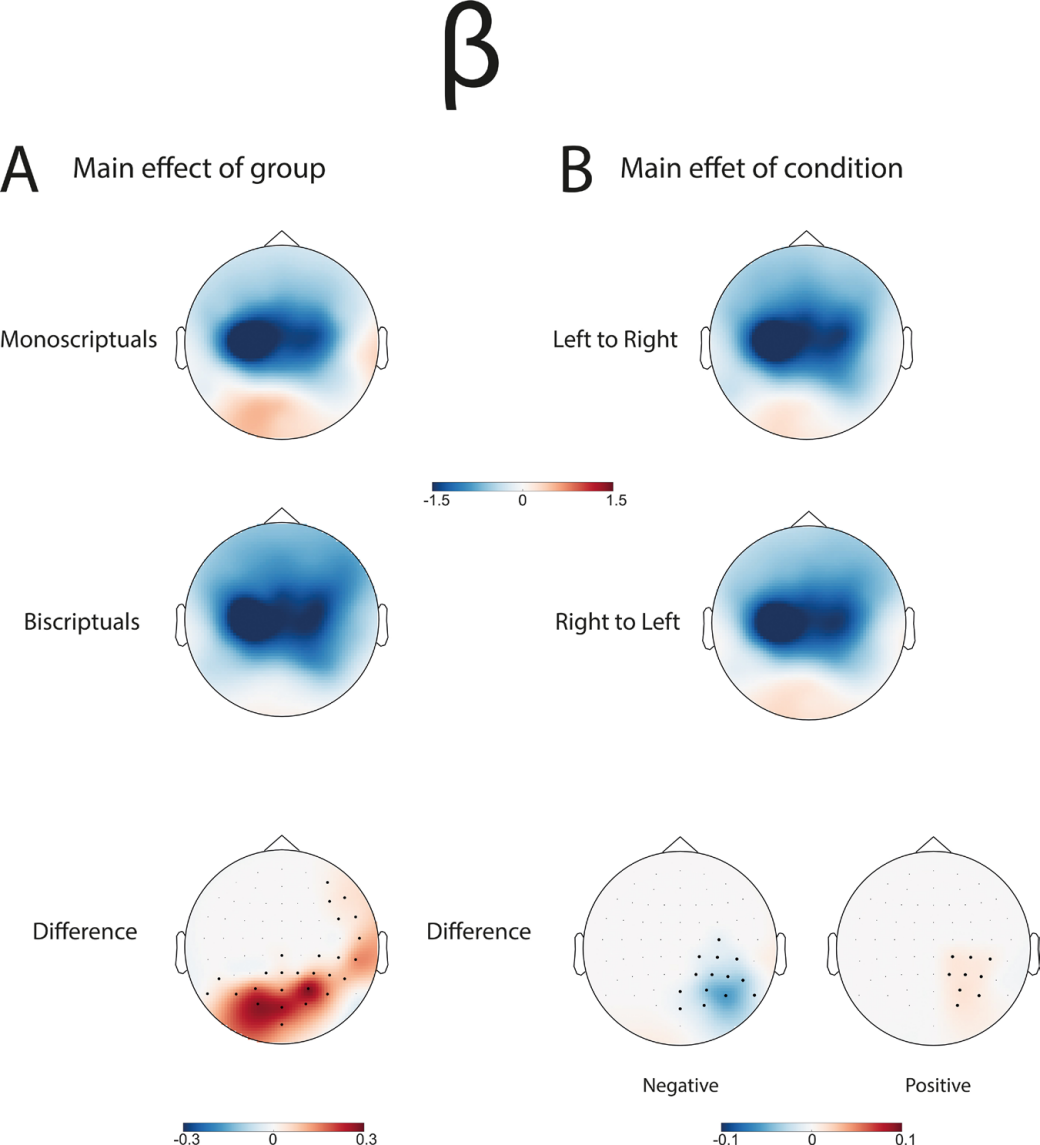
Beta power (13–30 Hz) during loops tracing. (A) Main effect of group on beta power. From top to bottom: beta power averaged across monoscriptuals; beta power averaged across biscriptuals; the significant cluster issued from the group comparison of beta power. (B) Main effect of condition on beta power. From top to bottom: beta power averaged across all subjects for the left-to-right condition; beta power averaged across all subjects for the right-to-left condition; topographical maps of the significant (left) negative cluster at 19–21 Hz and (right) positive cluster at 13 Hz issued from the condition comparison of beta power. Black dots denote the electrodes that belong to significant clusters.

Loop tracing elicited modulation of beta-band activity (13–30 Hz) in both groups, characterized mainly by a strong desynchronization over central electrodes (Fig. 5A). While this central desynchronization did not differ between groups, the group comparison revealed a significant positive cluster (*p* < .041) for the entire beta range (13–30 Hz), which includes a wide range of parieto-occipital electrodes and peripheral electrodes over the right hemisphere. In this cluster, monoscriptuals exhibit principally stronger beta synchronization in the occipital electrodes than biscriptuals (Fig. 5A).

Comparison across writing conditions revealed two significant clusters (Fig. 5B). A negative cluster (*p* < .011) centered around occipital electrodes in the ispilateral (right) hemisphere, between 19–21 Hz, indicating stronger beta desynchronization in the left-to-right than in the right-to-left condition. Conversely, a smaller positive cluster (*p* < .014) at 13 Hz in the same region showed reduced beta desynchronization in the left-to-right condition. There was no significant interaction between group and condition.

**Fig. 6.**
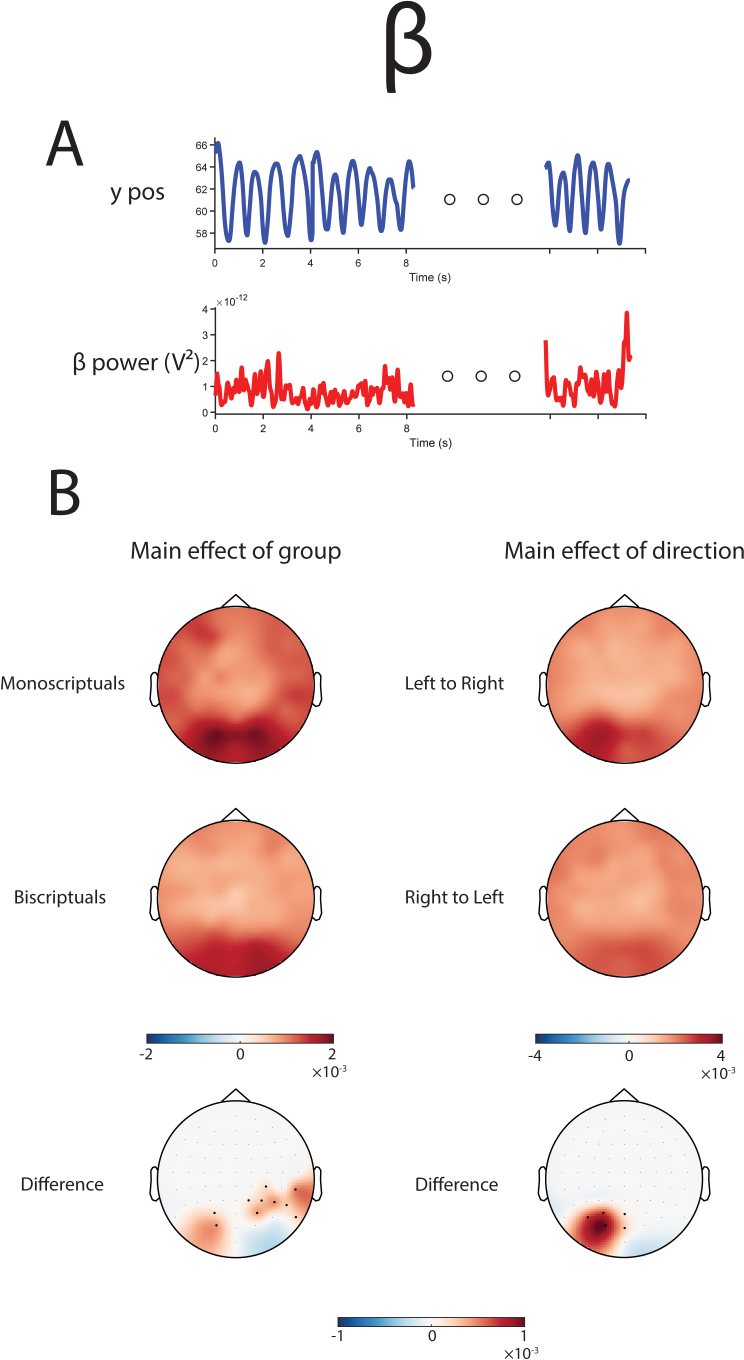
Cross-correlation between beta power and vertical pen position (y). (A) Example of the variation of y position and beta power (V²) of the O1 electrode as a function of time for trials that were concatenated for the analysis. (B) Main effect of (left) group and of (right) condition on the maximum cross-correlation. The topographical maps follow the same convention as in Figures 3 and 4. From top to bottom the averages of one group and condition (monosciptuals and left-to-right), then the averages of the other group and the other conditions (biscriptuals and right-to-left), and finally the significant clusters issued from group and condition comparisons are shown, respectively. Black dots denote the electrodes belonging to the significant clusters.

Beta activities might be associated with an ongoing dynamic state that co-varies with the continuously changing external variables, such as the visuospatial perception of the pen tip (Pei et al., 2021). We, thus, analyzed how beta power (13–30 Hz) covaried with participants’ movements, and more specifically with the vertical pen position (y; Fig. 6A) by cross correlating the amplitude of beta oscillations and y. Maximum correlation values were extracted for the subsequent statistical analysis. Two significant positive clusters were found when comparing monoscriptuals to biscriptuals, over right parieto-occipital (*p* < .023) and left occipital (*p* < .034) electrodes (Fig. 6B). Beta activity was more strongly correlated with the movement’s dynamics in monoscriptuals than in biscriptuals in these channels. Condition comparison also revealed a positive occipital cluster (*p* < .015) showing stronger beta–movement coupling in the left-to-right than in the right-to-left condition. Non-parametric tests on lag distributions showed no dominant temporal lag for either group, or a significant group difference. All the lags of maximum correlation did not differ significantly from zero in these significant clusters, indicating synchronous variations of beta power and movement dynamics. Overall, this shows that monoscriptuals exhibit stronger beta synchronization and tighter coupling between beta power and movement, over occipital regions.

## 6 Discussion

We assessed the neurophysiological mechanisms underpinning the biscriptual advantage in graphomotor coordination. We found that this behavioral advantage emerged even in the constrained EEG setup. At the neural level, it was associated with the decreased amplitude of both frequency bands targeted: theta (4–7 Hz) and beta (13–30 Hz). At the kinematic level, biscriptuals produced loops with significantly higher frequency and stability than monoscriptuals. Graphomotor coordination was also massively affected by the writing condition. Left-to-right loops were traced with higher frequency and higher stability than right-to-left loops. Their mean relative phase was also closer to the graphomotor pattern with a RP of 45 degrees, typical of a high level of performance (Alhaddad et al., 2023; Danna et al., 2011). Interaction effects further indicated that biscriptuals’ graphomotor coordination was less affected by the tracing condition than that of monoscriptuals. This finding fully replicates our previous observations of a biscriptual advantage in graphomotor coordination (Alhaddad et al., 2023, 2024) in a more constrained setting and with a larger number of trials. It thereby confirms the major impact of the type of writing expertise developed by writing in two distinct scripts on the motor processes allowing the planning and control of hand trajectories. At the neurophysiological level, monoscriptuals showed higher frontal theta power compared to biscriptuals. Frontal theta power was also positively correlated with behavioral variability. Monoscriptuals also displayed a higher degree of beta synchronization than biscriptuals over parieto-occipital electrodes, with beta power being more correlated with pen tip position dynamics. Similar effects in the beta band were observed for all participants when task difficulty increased, pointing towards a more general effect of task demands on beta activity. These effects suggest that both oscillatory components may contribute to the biscriptual advantage. However, only the optimization of predictive inference mediated by the effects of midfrontal theta oscillations stands out as a hallmark of the biscriptual advantage, because the effects observed in the beta band are also present when task difficulty increased.

### 6.1 Cost of planning and monitoring reflected by theta: A neural indicator for the biscriptual advantage

Our finding of a stronger theta activity during loop tracing in mono- than biscriptuals can be related to differences in planning and monitoring processes induced by the type of writing expertise. Theta synchronization in the frontal cortex has been considered to support processes broadly grouped under the terms of cognitive or executive control (Cavanagh & Frank, 2014). A prominent theta activity in the prefrontal cortex is induced by cognitive planning, in which a sequenced plan is developed and maintained to achieve a goal (Domic-Siede et al., 2021, 2023), especially during motor tasks (Domic-Siede et al., 2023; Pellegrino et al., 2018). The prefrontal cortex implements task rules, then computes an appropriate action to execute through a theta synchrony towards the motor cortex (Luu & Tucker, 2001; Weber et al., 2024), a coupling that strengthens under conditions with less available predictive information (Weber et al., 2024). The higher theta amplitude in mono- than biscriptuals could result from less accurate and efficient probabilistic inferences leading to a greater computational cost of generating and maintaining an appropriate action.

In addition to planning, midfrontal theta has been proposed to support a variety of subprocesses related to action monitoring (Cavanagh et al., 2012). It operates over different temporal scales during continuous actions (Cohen, 2016). Theta oscillations could convey a temporal frame of reference for the organization of distant neural processes, and may serve as a mechanism for communication between cortical sites involved in such control (Benedetto et al., 2021; Cavanagh & Cohen, 2022; Cavanagh et al., 2009, 2012). Furthermore, frontal theta power increases when ongoing actions are monitored under greater uncertainty to optimize behavior (Sandre & Weinberg, 2019). Accordingly, monoscriptuals who exhibit a lower level of mastery in graphomotor control and thus encounter greater uncertainty during the task would require a higher theta amplitude to exert effective control over movement execution.

Theta oscillations have also been shown to be related to motor stability (Cooper et al., 2017; Croce et al., 2022). In our task, the positive correlation between theta amplitude and behavioral variability is compatible with the interpretations outlined above, that theta amplitude appears to index the degree of control effort and the computational cost under a certain task demand, rather than optimal control per se. Continuous adjustments are constantly carried out through the lower-level sensorimotor network, and when these adjustments are insufficient to maintain a stable motor output, frontal midline structures at the higher level are recruited to supply additional computational resources. This theta activity might reflect the need to facilitate movements with various degrees of fluctuations moment-to-moment, hence mapping more onto unstable motor outcome.

Collectively, the biscriptual advantage might stem from a lower computational demand for planning, monitoring and stabilizing graphomotor movements. The absence of comparable effects across tracing conditions suggests that changes in task demand do not necessarily elicit equivalent variability in these mechanisms. Consequently, the decreased involvement of theta oscillations constitutes the neurophysiological signature of the biscriptual advantage.

### 6.2 Beta oscillations index various levels of reliance on visual feedback

The patterns of oscillatory power during the loop tracing task revealed a massive desynchronization in the beta-band across bilateral sensorimotor regions, more pronounced in the contralateral (left) side. This is in line with a previous view on the functionality of event-related beta desynchronization, which is considered to reflect the transition from the sensorimotor system’s idle state to its activation, as well as various sensory and cognitive processes involved in motor control (Engel & Fries, 2010; Kilavik et al., 2013). This validates our motor task as it, indeed, implicates a smooth motor control, and confirms that a strong and reliable beta desynchronization is clearly detectable in EEG signals during a continuous graphomotor task. More broadly, electrophysiological activities in the motor cortex are crucial for encoding movement trajectories during handwriting (Qi et al., 2025; Willett et al., 2021). In our study, we did not observe amplitude differences in beta desynchronization over the central region according to expertise or to tracing conditions. It is possible that this metric does not accurately capture the kinematics of the movements in our specific task (Kadmon Harpaz et al., 2014).

The effects were instead localized over the parieto-occipital region and characterized by beta synchronization rather than desynchronization, which alternatively, may stem from differences in sensory processing between groups and conditions. Beta oscillations have been hypothesized to additionally support and modulate sensory processing (Engel & Fries, 2010), by integrating current contextual state and external demands (Limanowski et al., 2020; Richter et al., 2018). Rhythmic predictive coding in visual processing could employ beta oscillations in the occipital cortex, which also carry visual information in a temporally predictive fashion (Han & VanRullen, 2017; Turner et al., 2023). Additionally, beta oscillations could support the integration of distinct perceptual elements through neural communication (Aissani et al., 2014). This is particularly relevant in our task, as the brain must continuously process visual information with high temporal resolution contained in the generated trace of movement via the magnocellular-dorsal visual stream, as well as somatosensory feedback, while simultaneously accounting for forward predictions derived from the efference copy. These predictions are less accurate in monoscriptuals as well as in the more difficult right-to-left condition, resulting in a heavier reliance on sensory feedback to complete the movements, evidenced by a higher beta synchronization in the posterior electrodes, as well as a higher degree of covariance of beta oscillations with the kinematics.

### 6.3 Possible cerebellar contributions to different types of expertise in graphomotor control

The effects observed in the beta band power and the beta-kinematic correlation in group and condition comparisons over the posterior electrodes might also stem from cerebellar contributions. Cerebellar activities are often overlooked in EEG studies. They are considerably more difficult to be captured in a traditional 64 electrodes EEG setup, partially due to the deep neuroanatomical position of the cerebellum and the ‘closed field’ signature of the cerebellar morphology (Bantli, 1972), and partially due to the lack of coverage of electrodes on the top of the neck (Andersen et al., 2020). However, this is not to be disregarded, since cerebellar activities should only be attenuated by 30-60% compared to cortical signal in EEG despite its highly convoluted structure (Samuelsson et al., 2020). In the predictive coding framework, cerebellum is considered as a key node that maintains the internal model and integrates predictions and online feedback of motor events (Kawato, 1999; Popa & Ebner, 2018; Wolpert et al., 1998), with kinematic parameters displaying strong coherence with its activity (Bourguignon et al., 2015). In the context of handwriting, cerebellum is strongly recruited and is sensitive to levels of expertise and task difficulties (Palmis et al., 2019, 2021). More specifically to the beta band, beta oscillations are functionally relevant in a cerebello-diencephalic-cortical network in motor control (Pollok et al., 2008), as well as to somatosensory inputs in the cerebellum (Andersen & Lundqvist, 2019; Tesche & Karhu, 2000). Given the physiological feasibility of recording electrophysiological activities of the cerebellum, its central role in graphomotor control, as well as its involvement in beta-band activity, the differences in beta synchronization amplitude and its correlation with movements may at least partially result from cerebellar contributions modulated by expertise and task difficulty.

### 6.4 Limitations and conclusions

There are several limitations to our study. While our study was not initially designed to tackle the sources of the neural processes, it might be worthy in the future to use specific electrode coverage designed to capture cerebellar activity and source localization methods, in order to discriminate cortical from cerebellar contributions of the observed beta band modulations. Additionally, how the cerebellum regulates the movements in a more global manner needs to be investigated. Indeed, pre-movement bursts of the beta oscillations in the cerebellum predict motor performance by integrating previous outcomes contextually (Bracco et al., 2026). Constraints of the generalizability are also of concern, since only Latin-Arabic biscriptuality in the expatriated subjects was tested, other types of practice of different scripts sharing different degrees of visuomotor characteristics thereby deserve further investigation using more various graphomotor tasks (Alhaddad et al., 2023, 2024; Kataoka et al., 2025; Tseng, 1998). Furthermore, even though the loop tracing task represents well the non-linear coupling of the two motor oscillators from the wrist and the fingers at play during cursive handwriting, it does not fully generalize to more sequential handwriting styles encompassing pen lifts. Tasks of handwriting in a linguistic context could be employed in the future, at the light of the current findings. Knowing that psycholinguistic features impact handwriting kinematics, this dimension needs to be carefully controlled in such future endeavors (Kandel & Perret, 2015; Palmis et al., 2019).

The biscriptual advantage on graphomotor coordination is extremely robust, as the present study is the third replication after Alhaddad et al. (2023, 2024). Writing practice, indeed, has a broader impact on manual motor function, mediated by an optimization of predictive inferences issued from the prefrontal cortex, as well as a shifting between the reliance on efference copy predictions and on sensory feedback. Biscriptuals have a reduced need of computational resources for action planning and monitoring, along with a higher confidence of the accuracy of the forward predictions of the movement outcomes, emphasizing the contributions from components that are both prefrontal and sensorimotor to implement precise internal predictive models to achieve an advantageous expertise. An essential parallel can be drawn with bilingualism, where experience managing two linguistic systems enhances neural functions that support efficient executive processing that support language learning and modulate proficiency in acquiring additional languages (Hayakawa & Marian, 2019). Moreover, bilinguals demonstrate more efficient cognitive control during language processing, requiring fewer resources to manage interference and select appropriate representations (Freeman et al., 2016). Collectively, these converging lines of evidence suggest that experience-dependent motor-based diversity, much like linguistic-based diversity, could act as an asset for optimizing the cognitive and sensorimotor systems that support skilled performance.

## Supplementary Material

Supplementary Material

## Data Availability

All the data and code in this study will be made available from the corresponding author upon reasonable requests.
