# Supplementary material for: Neurophysiological mechanisms of optimized graphomotor performance in biscriptuals
Source: Imaging Neurosci (Camb). 2026 Jun 5;4:IMAG.a.1265. doi: 10.1162/IMAG.a.1265 (PMC13245215; doi:10.1162/IMAG.a.1265)
Supplement: Supplementary Material [file IMAG.a.1265_supp.pdf]

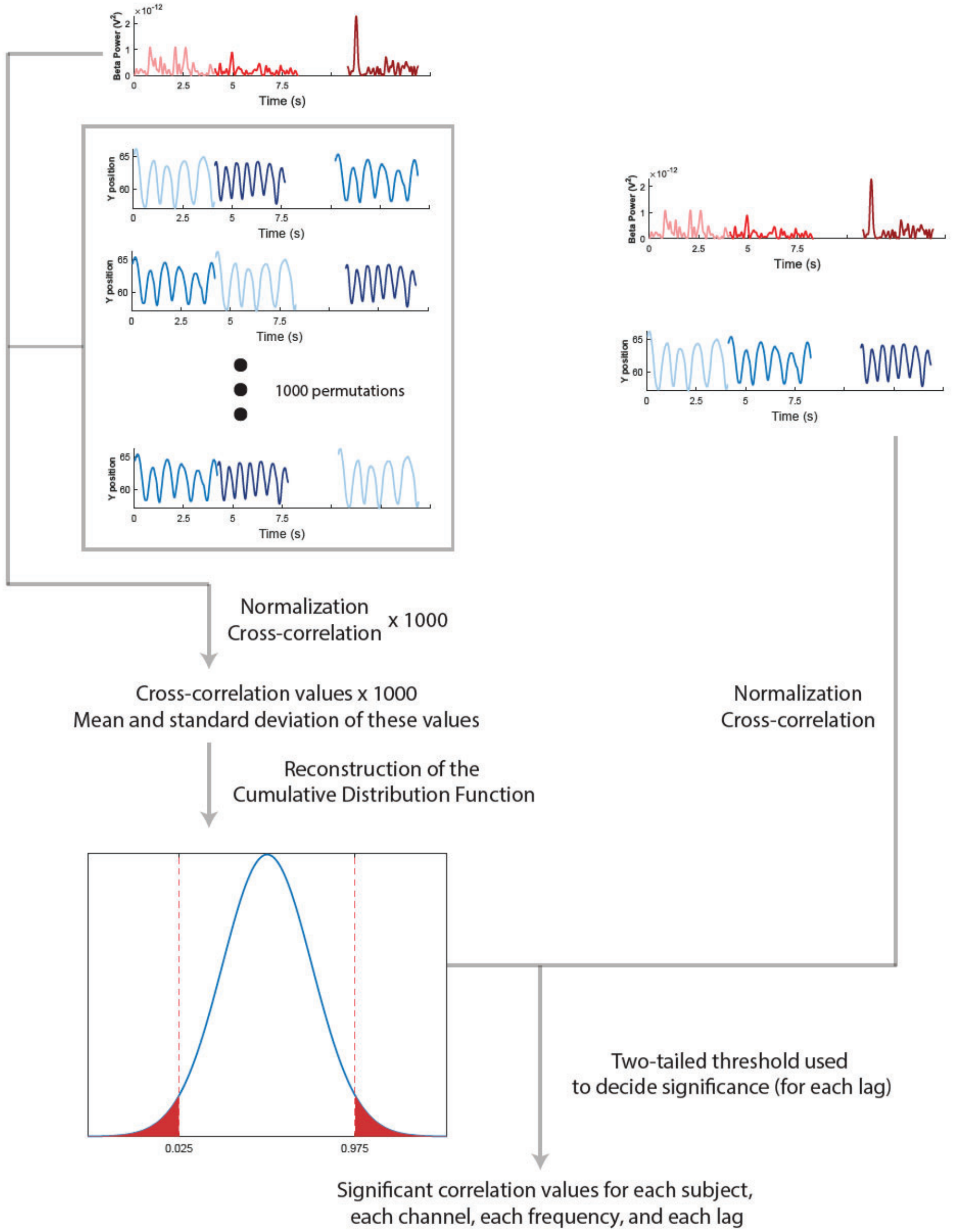

Supplementary Figure 1. Schematic representation of the pipeline to pre-threshold the correlation coefficients on the single-subject level. On the right: For each subject, each channel and each frequency, concatenated beta power and y position (208 for the group comparison, 104 for the condition comparison) are normalized and cross-correlated by a maximal lag of 200ms. On the left: the order of concatenation of the y position is permuted 1000 times, and each randomized permutation is normalized and cross-correlated with the normalized beta power in the same manner. The mean and the standard deviation of these cross-correlation results at each lag is used to construct a cumulative distribution function, with which the real correlation coefficient at one lag is compared. A two-tailed test is used for this comparison. For each subject, each channel, each frequency and each lag, a correlation coefficient is considered significant if it passes the two-tailed test in the calculated distribution function. The coefficients that do not meet this criterion are replaced by NaN.
